# Hypoxia induces pulmonary artery smooth muscle dysfunction through mitochondrial fragmentation-mediated endoplasmic reticulum stress

**DOI:** 10.18632/aging.103892

**Published:** 2020-11-18

**Authors:** Bing Zhuan, Xi Wang, Ming-Deng Wang, Zhi-Cai Li, Qun Yuan, Jun Xie, Zhao Yang

**Affiliations:** 1 Department of Respiratory Medicine, People’s Hospital of Ningxia Hui Autonomous Region, Yinchuan 750000, China; 2 Department of Respiratory Medicine, The Affiliated Suzhou Science and Technology Town Hospital of Nanjing Medical University, Suzhou 215153, China; 3 Department of Intensive Care Unit, The Affiliated Suzhou Science and Technology Town Hospital of Nanjing Medical University, Suzhou 215153, China; 4 Department of Thoracic Surgery, The Affiliated Suzhou Science and Technology Town Hospital of Nanjing Medical University, Suzhou 215153, China

**Keywords:** hypoxia, pulmonary arterial hypertension, mitochondrial fragmentation, ER stress

## Abstract

Pulmonary arterial hypertension (PAH) is characterized by pulmonary artery smooth muscle cell (PASMC) dysfunction. However, the underlying mechanisms of PASMC dysfunction remain largely unknown. Here, we show that mitochondrial fragmentation contributes to PASMC dysfunction through enhancement of endoplasmic reticulum (ER) stress. PASMC dysfunction accompanied by mitochondrial fragmentation and ER stress was observed in the pulmonary arteries of hypoxia-induced rats with PAH, as well as isolated PASMCs under hypoxia. Treatment with Mdivi-1 inhibited mitochondrial fragmentation and ER stress and improved PASMC function in isolated PASMCs under hypoxia, while Drp1 overexpression increased mitochondrial fragmentation and ER stress, impairing PASMC function in isolated PASMCs under normoxia. However, inhibition of ER stress using ER stress inhibitors showed a negligible effect on mitochondrial morphology but improved PASMC function during hypoxia. Additionally, we found that mitochondrial fragmentation-promoted ER stress was dependent on mitochondrial reactive oxygen species. Furthermore, inhibition of mitochondrial fragmentation using Mdivi-1 attenuated mitochondrial fragmentation and ER stress in hypoxic PASMCs and improved the pulmonary artery smooth muscle function in hypoxic rats. These results suggest that hypoxia induces pulmonary artery smooth muscle dysfunction through mitochondrial fragmentation-mediated ER stress and that mitochondrial morphology is a potential target for treatment of hypoxia-induced pulmonary artery smooth muscle dysfunction.

## INTRODUCTION

Pulmonary arteries irrigate pulmonary alveoli, where gas exchange takes place between the bloodstream and the lungs. In contrast to other vasculatures, pulmonary arteries constrict in response to hypoxia to distribute blood away from poorly oxygenated alveoli [1, 2]. As a result, chronic hypoxia increases pulmonary artery smooth muscle cell (PASMC) proliferation and dysfunction, leading to a narrowing of pulmonary circulation, poor blood and tissue oxygenation, and an increased workload in the right portion of the heart [3, 4]. This mechanism is relevant for conditions such as pulmonary arterial hypertension (PAH), a chronic and incurable disease. PAH is characterized by increased pulmonary arterial blood pressure and right ventricular hypertrophy [5–7]. The course of PAH progresses rapidly and ultimately leads to right ventricular failure and premature death. Thus, understanding the underlying mechanisms involved in PAH should help design specific and efficient therapies for this life-threatening disease.

Evidence has shown that vascular metabolism is largely dependent on glycolysis, even under normal, well-oxygenated conditions, to supply ATP and sustain a variety of cell functions [8, 9]. However, the role of mitochondria in vascular function remains largely unexplored. Although the contribution of mitochondria to vascular smooth muscle cell bioenergetics is low, several lines of evidence highlight the relevance of mitochondria in the control of their function. Mitochondrial motility and dynamics are reported to be involved in the regulation of the capacity for contraction, proliferation, migration, and secretion in vascular smooth muscle cells, and mitochondrial dysfunction can result in vascular smooth muscle cell phenotypic switch which promotes vascular pathologies such as atherosclerosis, stenosis, and hypertension [10–12]. The role of the mitochondria in PAH, however, remains largely unknown. Different from other organelles, mitochondria form a complex interconnected network that undergoes continuous fusion and fission. Mitochondrial fusion is controlled by mitofusin (mfn) 1, mfn 2, and optic atrophy 1 (OPA1), while mitochondrial fission is controlled by the GTPase dynamin-related protein 1 (Drp1) and mitochondrial fission 1 (fis1). Mitochondrial fragmentation results from an imbalanced mitochondrial fission/fusion, and was observed in PASMCs in hypoxia-induced PAH [4], indicating a potential role of mitochondrial dynamics in the pathological process of PAH.

Here, we tested the role of mitochondrial dynamics in the development of PASMC dysfunction in hypoxia-induced PAH. We found that mitochondrial fragmentation is a cause of PASMC dysfunction through enhancement of endoplasmic reticulum (ER) stress in hypoxia-induced PAH. These findings suggested that mitochondrial fragmentation is a potential target for treatment of PAH.

## RESULTS

### Hypoxia induced pulmonary artery SMC dysfunction

**Figure 1:**
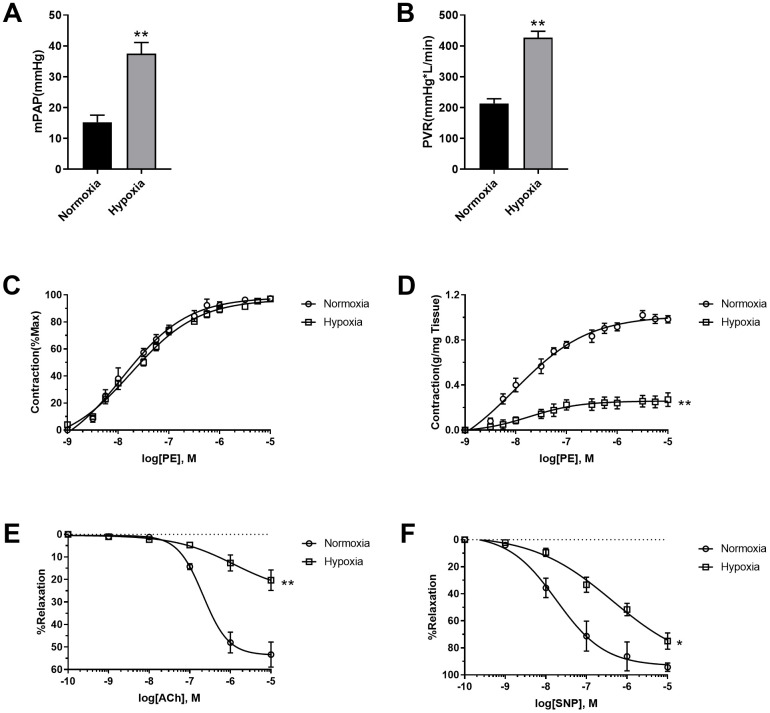
**Hypoxia-induced PASMC dysfunction.** (**A**) Hypoxia increased mean pulmonary artery pressure (mPAP) in rats. (**B**) Hypoxia increased pulmonary vessel resistance (PVR) in rats. (**C**) PE-induced vasoconstriction presented as percentage of maximal contraction. (**D**) PE-induced vasoconstriction presented as contraction force per tissue weight (g/mg tissue). (**E**, **F**) Both endothelium-dependent (**E**) and endothelium-independent (**F**) vasodilation was impaired in hypoxic rats. *, *p*<0.05, **, *p*<0.01. n=8.

To assess the effects of hypoxia on pulmonary vascular function, mean pulmonary artery pressure (mPAP) and Pulmonary vessel resistance (PVR) were detected. As shown in Figure 1A, 1B, hypoxia increased both mPAP and PVR, which are typical characteristics of PAH pathogenesis. The pulmonary arteries were then isolated and their function was detected. Although PE seemed to be equally potent in the stimulation of pulmonary vasoconstriction in normoxic and hypoxic rats when PE-induced vasoconstriction was presented as percentage of maximal contraction (Figure 1C), PE-induced vasoconstriction was reduced in pulmonary artery rings in hypoxic rats when it was presented as contraction force per tissue weight (g/mg tissue) (Figure 1D), suggesting the occurrence of PASMC dysfunction. That notion has been confirmed by vascular functions of vasodilation. Both endothelium-dependent (acetylcholine (Ach)-induced) and endothelium-independent (sodium nitroprusside (SNP)-induced) vasodilation were impaired in hypoxic rats compared to normoxic rats (Figure 1E, 1F). These results suggested that both pulmonary artery endothelial cells and PASMCs were dysfunctional in hypoxic rats.

### Hypoxia induced mitochondrial fragmentation and ER stress in PASMCs

**Figure 2:**
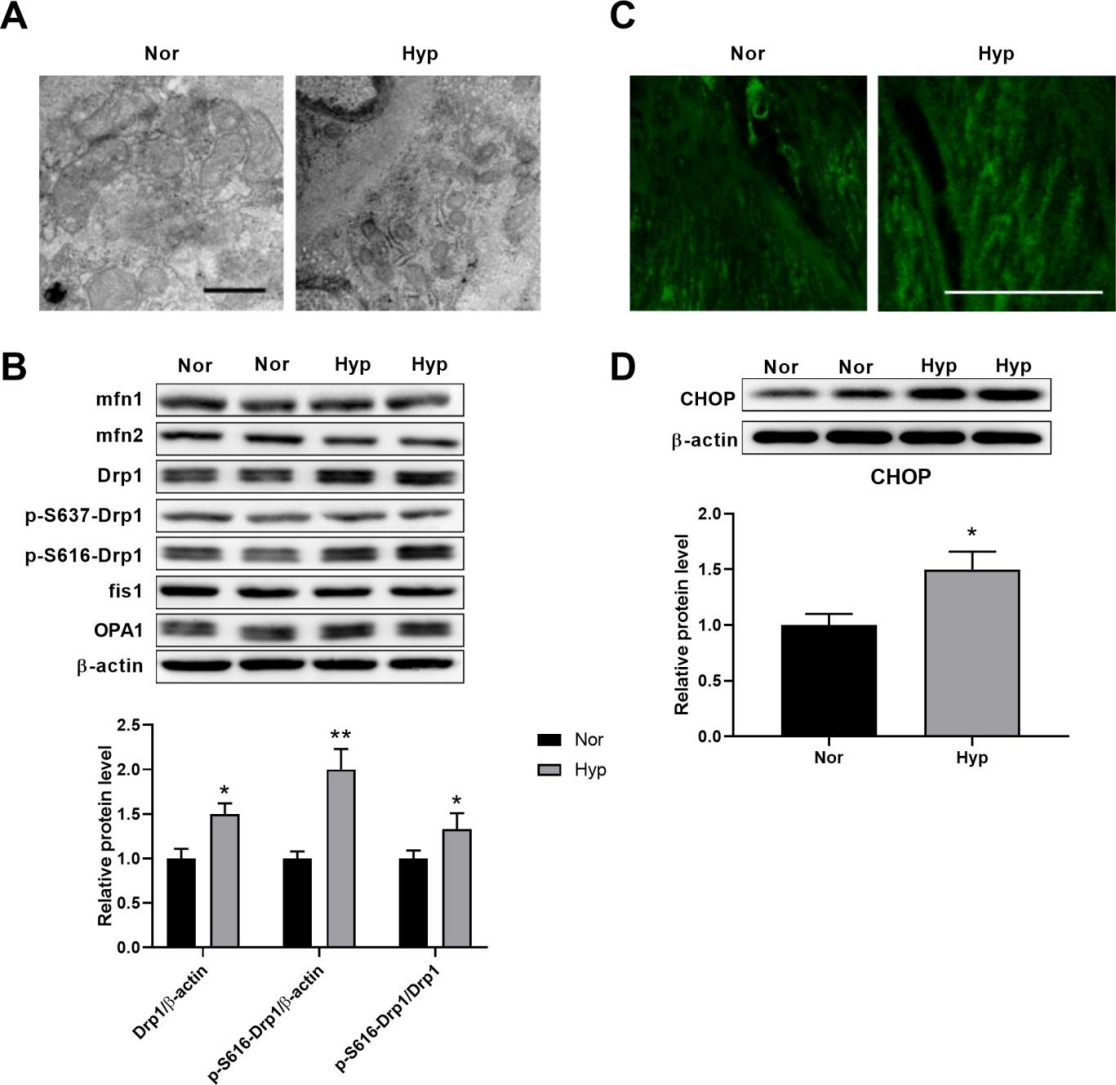
**Hypoxia induced mitochondrial fragmentation and ER stress in PASMCs.** (**A**) Ultrastructural alteration of the mitochondria and ER in pulmonary artery smooth muscle of normoxic and hypoxic rats. Scale bar, 500 nm. (**B**) Expression of mitochondrial dynamics-related proteins in endothelium-denuded pulmonary arteries. Twenty micrograms of protein was loaded in each lane. (**C**) Mitochondrial ROS detected by mitoSOX in isolated pulmonary arteries. Scale bar, 20 μm. (**D**) ER stress as assessed by CHOP expression in endothelium-denuded pulmonary arteries. Twenty micrograms of protein was loaded for each lane. *, *p*<0.05, **, *p*<0.01. n=8.

Transmission electron microscopy was then used to test the ultrastructural alterations of mitochondria in the PASMCs of hypoxic rats. As shown in Figure 2A, hypoxia-induced mitochondrial fragmentation was characterized by an increased mitochondrial number and decreased mitochondrial size in the PASMCs of hypoxic rats. To examine the underlying mechanisms of mitochondrial fragmentation, we next detected the contents of the major mitochondrial dynamics-related proteins in endothelium-denuded pulmonary arteries. No differences in the contents of mfn1, mfn2, OPA1, and fis1 were detected in hypoxic rats (Figure 2B). Specifically, the levels of Drp1 and Drp1 phosphorylation at serine 616 were increased and Drp1 phosphorylation at serine 637 showed little alteration in hypoxic rats. It has been observed that Drp1 phosphorylation at serine 616 promotes mitochondrial fission, while Drp1 phosphorylation at serine 637 inhibits mitochondrial fission [16], suggesting that Drp1 phosphorylation at serine 616 may contribute to mitochondrial fragmentation in PASMCs in hypoxic rats. Mitochondrial fragmentation was accompanied by an increased mitochondrial reactive oxygen species (ROS) as detected by mitoSOX (Figure 2C). In addition, a significant ultrastructural alteration of ER was observed in pulmonary aortic SMCs (Figure 2A). The ER was disordered, indicating the existence of ER stress associated with various pathological processes. CHOP, which is activated by ER stress, was then detected. CHOP expression was increased in endothelium-denuded pulmonary arteries from hypoxic rats (Figure 2D).

### Mitochondrial fragmentation enhanced ER stress in cultured PASMCs under hypoxia

**Figure 3:**
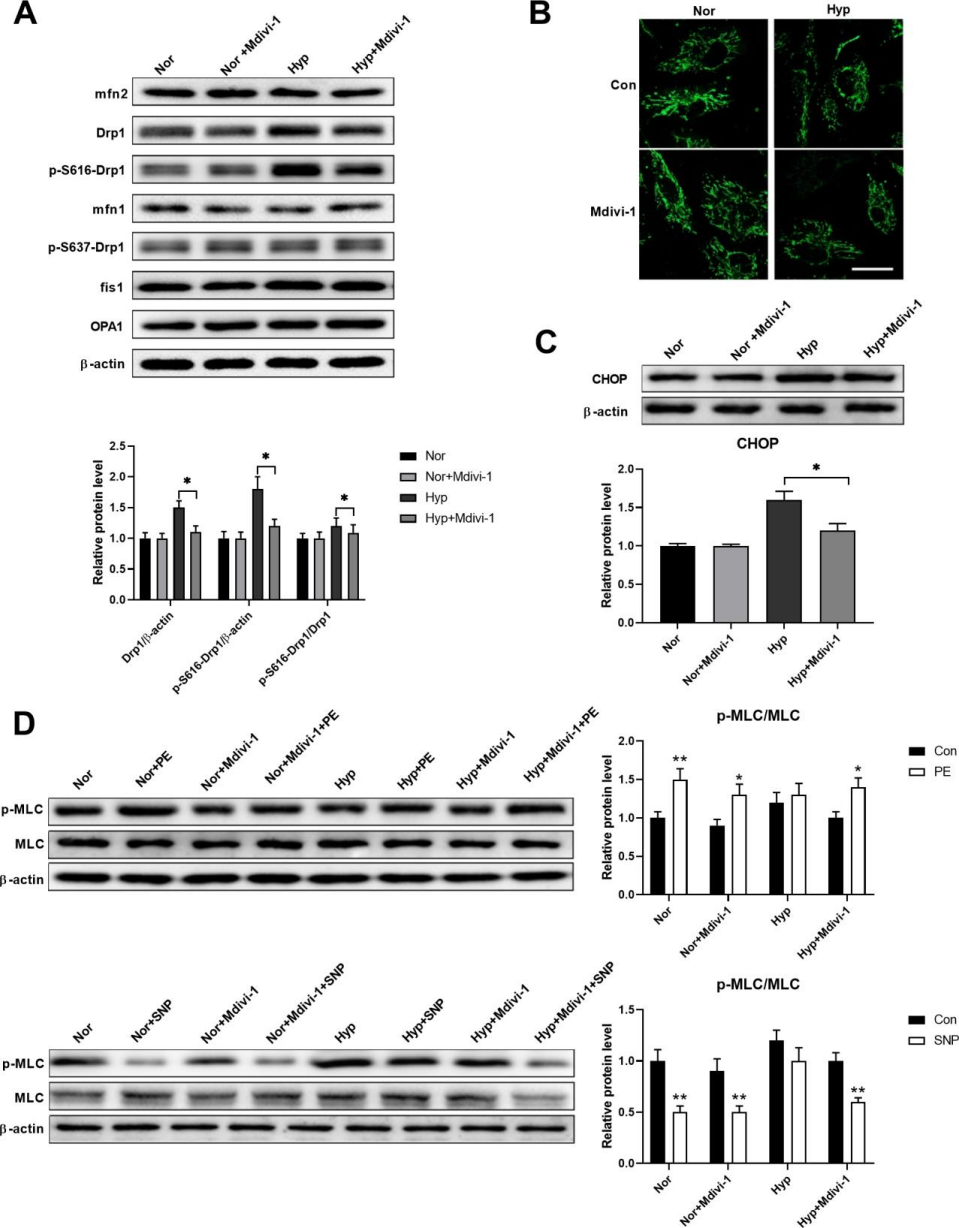
**Mitochondrial fragmentation enhanced ER stress in cultured PASMCs under hypoxia.** (**A**) Mdivi-1 treatment decreased Drp1 and Drp1 phosphorylation at serine 616 in PASMCs under hypoxia. Twenty micrograms of protein was loaded for each lane. (**B**) Mdivi-1 treatment inhibited mitochondrial fragmentation in PASMCs under hypoxia. Scale bar, 20 μm. (**C**) Mdivi-1 treatment inhibited ER stress as evidenced by the decreased CHOP expression in PASMCs under hypoxia. Twenty micrograms of protein was loaded for each lane. (**D**) Mdivi-1 treatment improved PASMC function as evidenced by increased PE/SNP-induced MLC phosphorylation/dephosphorylation in PASMCs under hypoxia. Twenty micrograms of protein was loaded for each lane. *, *p* < 0.05, **, *p* < 0.01. n = 8.

Studies have shown that ER stress and mitochondrial damage are closely linked [17, 18]. Thus, we next examined whether these two processes have crosstalk. A Drp1-specific inhibitor Mdivi-1 (20 μM) was used to inhibit mitochondrial fragmentation in isolated PASMCs. As shown in Figure 3A, hypoxia specifically increased Drp1 and Drp1 phosphorylation at serine 616, while it showed little effect on the other mitochondrial dynamics-related proteins. Mdivi-1 treatment showed little effects on all mitochondrial dynamics-related proteins in PASMCs under normoxia, except for a decreased Drp1 and Drp1 phosphorylation at serine 616 in PASMCs under hypoxia. In addition, hypoxia-induced mitochondrial fragmentation and Mdivi-1 treatment inhibited mitochondrial fragmentation in PASMCs under hypoxic conditions (Figure 3B). Inhibition of mitochondrial fragmentation also inhibited ER stress as evidenced by a decreased CHOP expression in PASMCs under hypoxia (Figure 3C). Furthermore, inhibition of mitochondrial fragmentation also improved PASMC function as evidenced by the increased PE/SNP-induced MLC phosphorylation in PASMCs under hypoxia (Figure 3D). These results suggested that Drp1-mediated mitochondrial fragmentation may enhance ER stress in PASMCs under hypoxia.

### Drp1 overexpression induced ER stress

**Figure 4:**
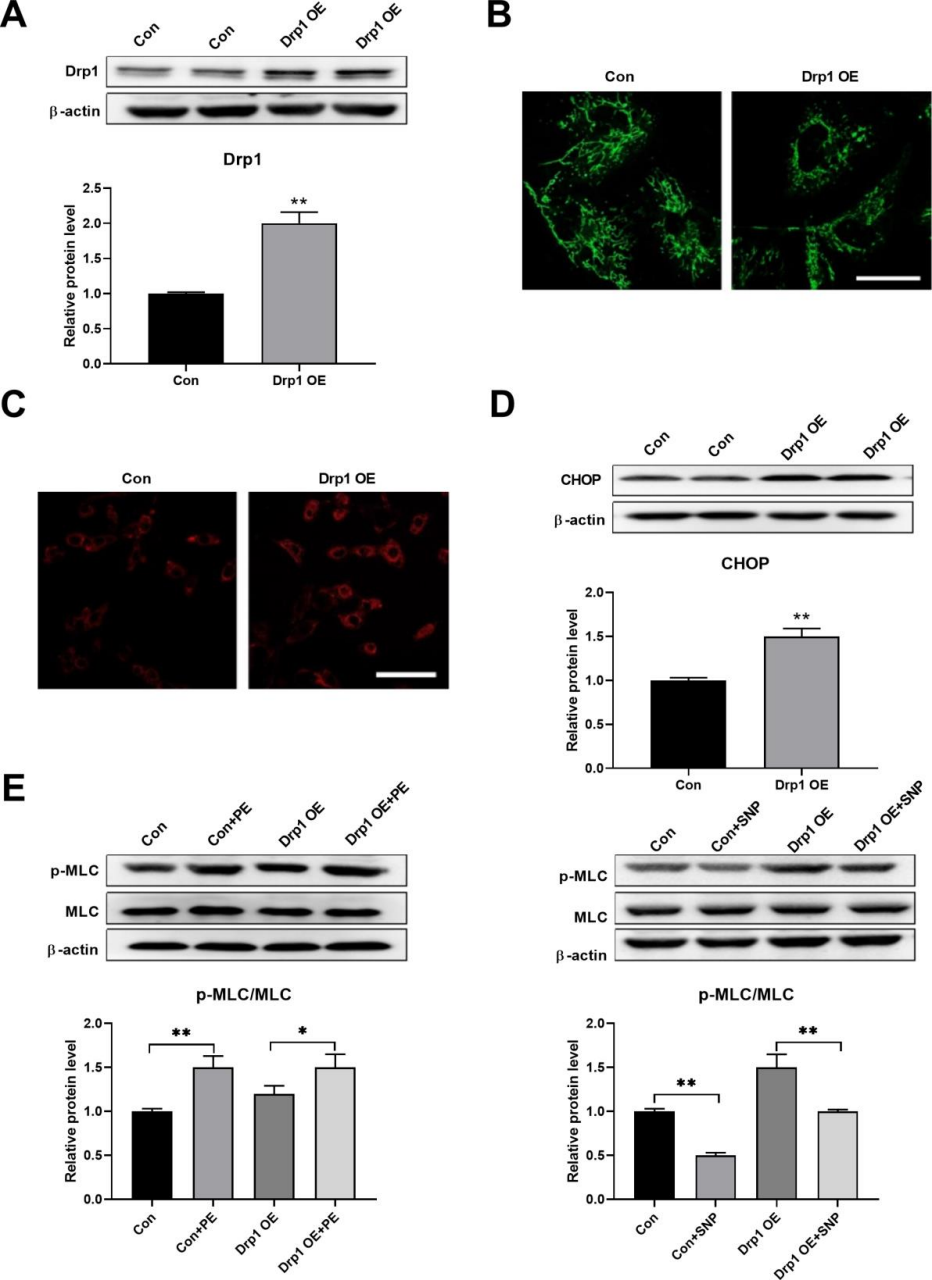
**Drp1 overexpression induced ER stress.** (**A**) Drp1 was overexpressed by adenovirus in PASMCs. Twenty micrograms of protein was loaded for each lane. (**B**) Overexpression of Drp1 induced mitochondrial fragmentation as detected by mitoTracker. Scale bar, 20 μm. (**C**) Overexpression of Drp1 increased mitochondrial ROS as detected by mitoSOX. Scale bar, 100 μm. (**D**) Drp1 overexpression increased CHOP expression in PASMCs. Twenty micrograms of protein was loaded for each lane. (**E**) Drp1 overexpression decreased PASMCs function as evidenced by decreased PE/SNP-induced MLC phosphorylation/dephosphorylation in PASMCs. Twenty micrograms of protein was loaded in each lane. *, *p* < 0.05, **, *p* < 0.01. n = 8.

Drp1 was then overexpressed to test whether mitochondrial fragmentation can induce ER stress directly. Drp1 was overexpressed using adenoviruses in PASMCs (Figure 4A). An overexpression of Drp1 induced mitochondrial fragmentation, as detected by mitoTracker (Figure 4B), and increased mitochondrial ROS, as detected by mitoSOX (Figure 4C). In addition, Drp1 overexpression increased CHOP expression in PASMCs, indicating an increase in ER stress (Figure 4D). Meanwhile, Drp1 overexpression also decreased PASMC function as evidenced by a decreased PE/SNP-induced MLC phosphorylation (Figure 4E). These results suggested that mitochondrial fragmentation can induce ER stress in PASMCs.

### Inhibition of ER stress showed little effects on mitochondrial morphology but improved cell function in PASMCs under hypoxia

**Figure 5:**
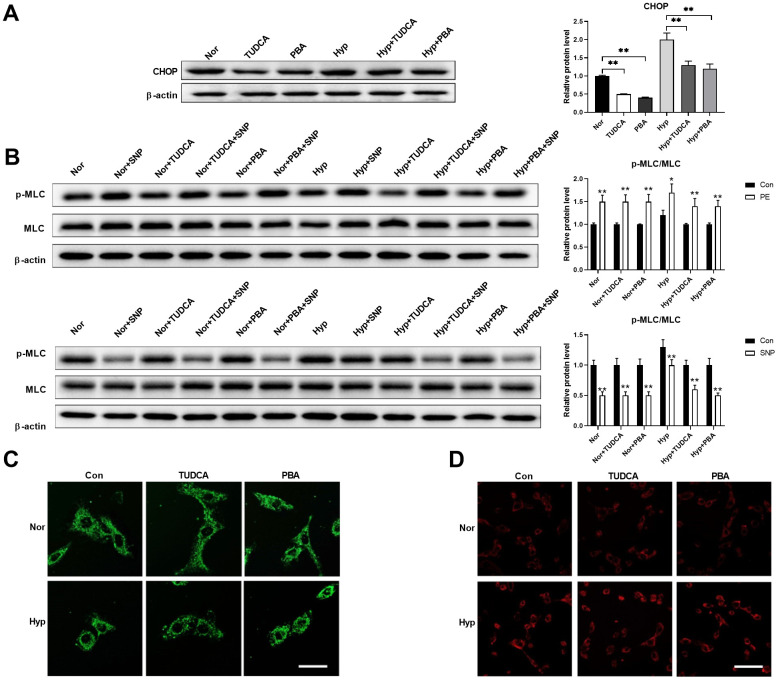
**Inhibition of ER stress showed little effects on mitochondrial morphology but improved cell function in PASMCs under hypoxia.** (**A**) PBA and TUDCA decreased CHOP expression in PASMCs under both normoxia and hypoxia. Twenty micrograms of protein was loaded in each lane. (**B**) PBA and TUDCA improved PASMC function as evidenced by increased PE/SNP-induced MLC phosphorylation in PASMCs under hypoxia. Twenty micrograms of protein was loaded in each lane. (**C**, **D**) PBA and TUDCA showed little effects on mitochondrial morphology (**C**) and mitochondrial ROS (**D**) in PASMCs under hypoxia. Scale bar, 20 μm in **C** and 100 μm in **D**. *, *p* < 0.05, **, *p* < 0.01. n = 8.

Next, we tested whether inhibition of ER stress using ER stress inhibitors affects mitochondrial morphology in PASMCs under hypoxia. Chemical or pharmaceutical chaperones, such as 4-phenyl butyric acid (PBA) and tauroursodeoxycholic acid (TUDCA), are frequently used ER stress inhibitors [19]. Both PBA (50 μM) and TUDCA (100 μM) decreased CHOP expression in PASMCs under both normoxic and hypoxic conditions (Figure 5A). In addition, inhibition of ER stress improved PASMC function as evidenced by an increased PE/SNP-induced MLC phosphorylation in PASMCs under hypoxia (Figure 5B). However, inhibition of ER stress showed little effects on mitochondrial morphology and mitochondrial ROS in PASMCs under hypoxia (Figure 5C, 5D). These results suggested that inhibition of ER stress showed little effects on mitochondrial morphology, but improved cell function in PASMCs in hypoxic conditions.

### Mitochondrial ROS mediates the interaction between mitochondria and ER

**Figure 6:**
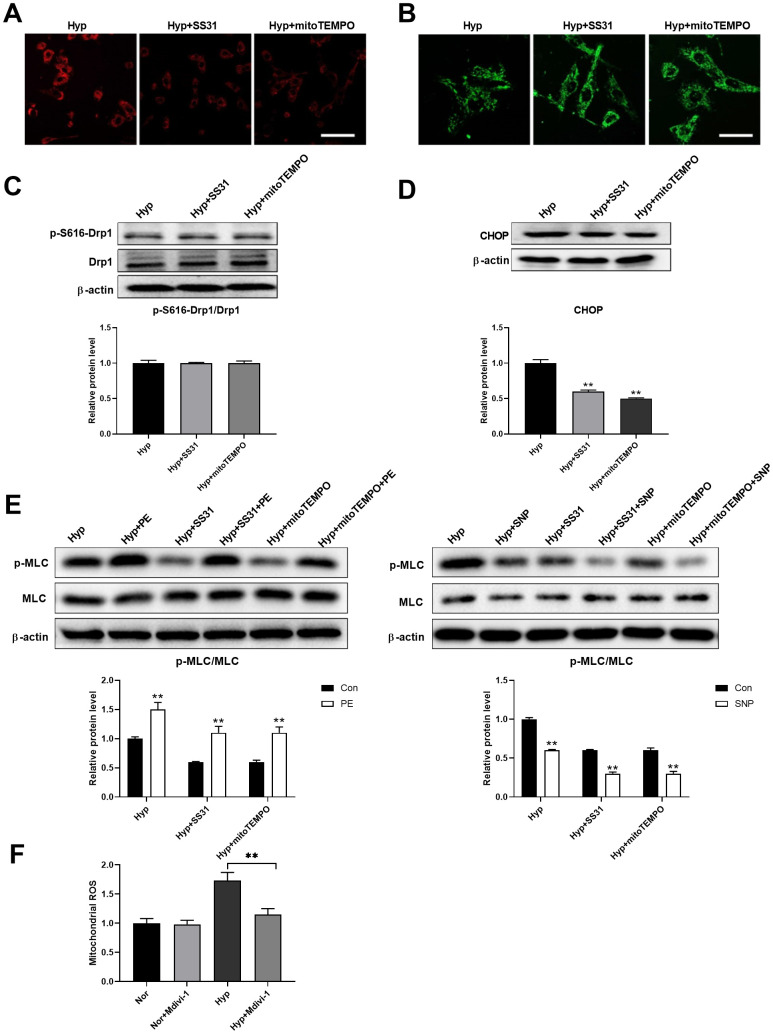
**Mitochondrial ROS mediated the interaction between mitochondria and ER.** (**A**) SS31 and mitoTEMPO scavenged mitochondrial ROS as detected by mitoSOX. Scale bar, 100 μm. (**B**) SS31 and mitoTEMPO inhibited hypoxia-induced mitochondrial fragmentation in PASMCs. Scale bar, 20 μm. (**C**) SS31 and mitoTEMPO showed little effects on Drp1 expression and Drp1 phosphorylation at serine 616 in PASMCs under hypoxia. Twenty micrograms of protein was loaded in each lane. (**D**) SS31 and mitoTEMPO decreased CHOP expression in PASMCs under hypoxia. Twenty micrograms of protein was loaded in each lane. (**E**) SS31 and mitoTEMPO improved PASMC function as evidenced by increased PE/SNP-induced MLC phosphorylation/dephosphorylation in PASMCs under hypoxia. Twenty micrograms of protein was loaded in each lane. (**F**) Inhibition of mitochondrial fragmentation using Mdivi-1 decreased mitochondrial ROS in PASMCs in hypoxia. *, *p* < 0.05, **, *p* < 0.01. n = 8.

Studies have shown that mitochondrial ROS plays an important role in the regulation of mitochondria-ER interactions [20–22]. We next tested the role of mitochondrial ROS in the regulation of ER stress. SS31 and mitoTEMPO, two classic mitochondrial ROS scavengers, were used to scavenge mitochondrial ROS (Figure 6A). Both SS31 and mitoTEMPO inhibited hypoxia-induced mitochondrial fragmentation in PASMCs (Figure 6B). Although both SS31 and mitoTEMPO showed little effects on Drp1 expression and Drp1 phosphorylation at serine 616, it decreased CHOP expression in PASMCs under hypoxia (Figure 6C, 6D). In addition, they also improved PASMC function as evidenced by increased PE/SNP-induced MLC phosphorylation in PASMCs under hypoxia (Figure 6E). Furthermore, inhibition of mitochondrial fragmentation using Mdivi-1 decreased mitochondrial ROS in PASMCs in hypoxic conditions (Figure 6F). These results suggested that mitochondrial ROS mediated mitochondrial fragmentation-induced ER stress.

### Inhibition of mitochondrial fragmentation using Mdivi-1 improved pulmonary artery smooth muscle function in response to hypoxia *in vivo*

**Figure 7:**
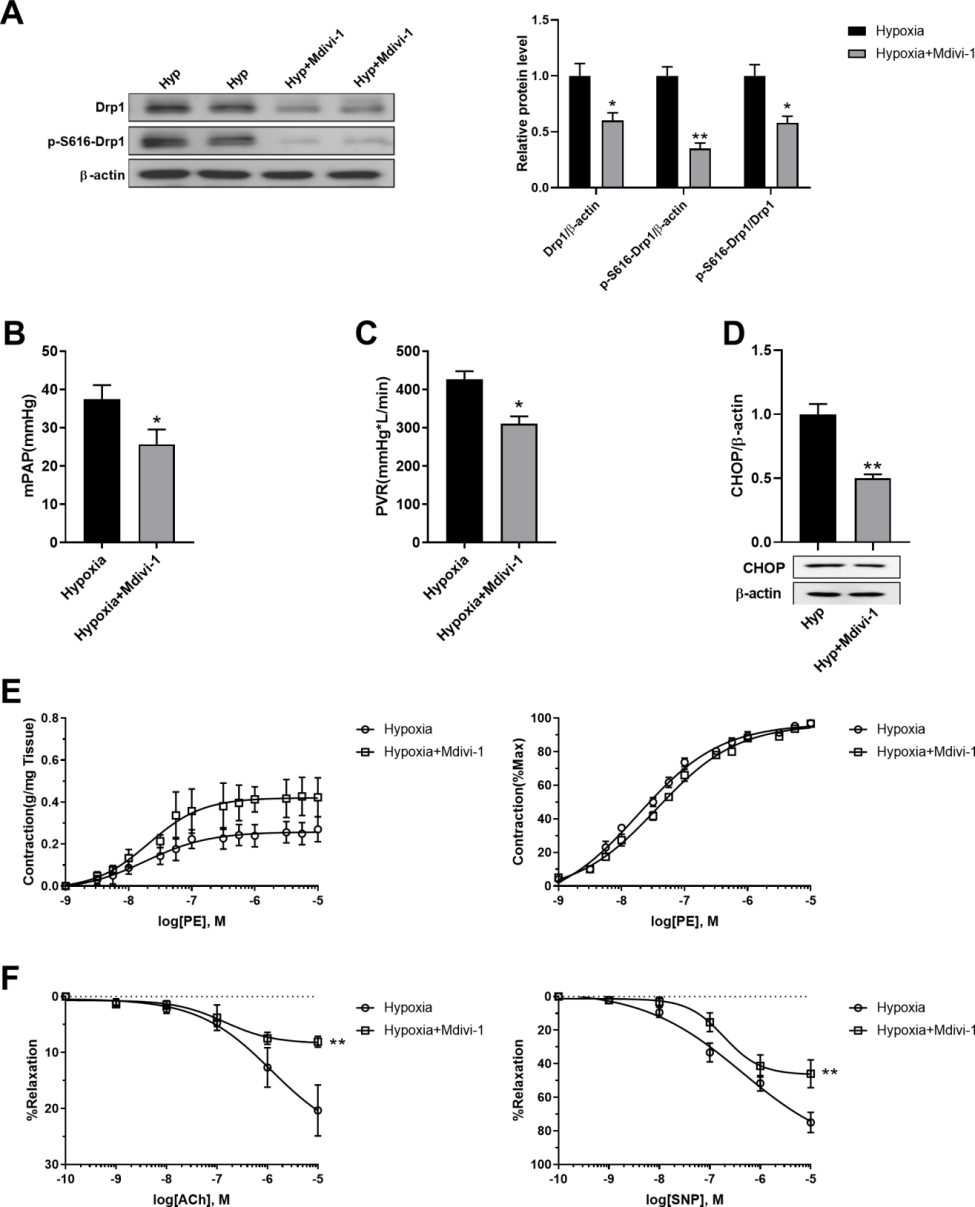
**Inhibition of mitochondrial fragmentation using Mdivi-1 improved pulmonary artery smooth muscle function in response to hypoxia *in vivo*.** (**A**) Mdivi-1 treatment decreased Drp1 and Drp1 phosphorylation at serine 616 in PASMCs of hypoxic rats. Twenty micrograms of protein was loaded in each lane. (**B**, **C**) Mdivi-1 treatment decreased both mPAP and PVR in hypoxia rats. (**D**) Mdivi-1 treatment deceased ER stress as detected by CHOP in isolated endothelium-denuded pulmonary arteries from hypoxic rats. Twenty micrograms of protein was loaded in each lane. (**E**, **F**) Mdivi-1 treatment improved PE-induced vasoconstriction (**E**) and ACh/SNP-induced vasodilation (**F**) in isolated pulmonary arteries from hypoxic rats. *, *p* < 0.05, **, *p* < 0.01. n = 8.

Mdivi-1 was then used to inhibit mitochondrial fragmentation in PASMCs of hypoxic rats *in vivo*. Mdivi-1 treatment decreased Drp1 and Drp1 phosphorylation at serine 616 in the PASMCs of hypoxic rats (Figure 7A). As a result, Mdivi-1 treatment decreased both mPAP and PVR in hypoxic rats (Figure 7B, 7C). In addition, it decreased ER stress, as detected by CHOP, and improved PE-induced vasoconstriction and ACh/SNP-induced vasodilation in isolated pulmonary arteries from hypoxic rats (Figure 7D, 7F). These results further confirmed that mitochondrial fragmentation in PASMCs contributed to pulmonary artery smooth muscle dysfunction, at least partly, through enhancement of ER stress in hypoxia.

## DISCUSSION

Although the mitochondria have been extensively studied in various physiological and pathological processes, their roles in vascular function remain largely unknown. Here, we found that mitochondrial fragmentation contributes to PASMC dysfunction through enhancement of ER stress. Inhibition of mitochondrial fragmentation using Mdivi-1 attenuated mitochondrial fragmentation and ER stress, improving PASMC function both *in vitro* and *in vivo*. In addition, we found that mitochondrial fragmentation-induced ER stress was dependent on mitochondrial ROS. These results suggested that mitochondrial morphology is a potential target for the treatment of hypoxia-induced pulmonary artery smooth muscle dysfunction.

Vascular cells contain less mitochondria, and they are mostly dependent on glycolysis for ATP supply. However, the role of mitochondria in vascular function remains largely unexplored. Here, we reported that mitochondrial fragmentation was observed in the PASMCs of hypoxic rats, which was consistent with previous studies [4, 23]. Mitochondrial morphology is delicately regulated by fusion and fission, both of which are essential in the regulation of cellular function. Mitochondrial fusion is associated with a distribution of metabolites, proteins and mtDNA, and the maintenance of cellular electrical and biochemical connectivity. Mitochondrial fission plays prominent roles in regulating autophagy, repair, cell division, and other processes. Dysregulation of mitochondrial dynamics has emerged as an important contributor to mitochondrial and vascular dysfunction in a variety of pathological conditions [24–26]. A previous study showed that hypoxia-induced mitochondrial fragmentation is associated with decreased expressions of peroxisome proliferator-activated receptor γ (PPARγ) and PPARγ co-activator 1α [23]. Here, we found that mitochondrial fragmentation was associated with hypoxia-induced Drp1 overexpression and an increase in Drp1 phosphorylation at serine 616 in the PASMCs of hypoxic rats. Inhibition of Drp1 using Mdivi-1 attenuated mitochondrial fragmentation and improved pulmonary artery function in hypoxic rats, suggesting that mitochondrial morphology plays important roles in the regulation of vascular function in PAH.

The mitochondria and ER are essential organelles in eukaryotic cells, which play key roles in various cellular functions. Recent advances have shown that these organelles communicate via the mitochondrial-associated ER membrane (MAM) to regulate various processes including bioenergetics, lipid traffic, Ca^2+^ buffering, and apoptosis [27–29]. Although the molecular composition of the MAM has not been fully understood, mounting evidence has shown that ER-mitochondrial interaction plays important roles in the regulation of various biological processes. Impaired ER-mitochondrial interactions may lead to metabolic disorders, cancers, and neurodegenerative diseases [30, 31]. Here, we showed that the inhibition of mitochondrial fragmentation using Mdivi-1 attenuates ER stress, and that inhibition of ER stress using PBA and TUDCA does not affect mitochondrial morphology, suggesting that mitochondrial fragmentation induces ER stress, while ER stress shows no effect on mitochondrial morphology, at least in hypoxic PASMCs. Although inhibition of ER stress showed no effect on mitochondrial morphology, it improved PASMC function in response to hypoxia. These results suggested that mitochondrial fragmentation induces PASMC dysfunction through the stimulation of ER stress. Although we still do not know whether such interaction is dependent on the MAM, it reinforced the notion that ER-mitochondrial interactions play important roles in the regulation of PASMC function in hypoxia.

Studies have shown that ROS mediates several critical aspects of ER stress in various conditions [32]. The ROS that mediates ER stress is either local, such as Nox4, an ER resident capable of producing ROS, or from other sources. In addition, ER stress also produces a secondary rise in ROS which induces adverse effects in cell function or survival [33, 34]. A recent study has shown that mitochondrial ROS is also involved in the induction of ER stress [22]. Here, the inhibition of mitochondrial ROS using SS31 and mitoTEMPO showed little effect on Drp1 expression and Drp1 phosphorylation at serine 616, but decreased ER stress in PASMCs under hypoxia, suggesting that mitochondrial ROS contributes to ER stress. Although whether this regulation is dependent on MAM is unknown, it indicates that mitochondrial ROS plays an important role in regulation of ER stress. The mechanism by which mitochondrial ROS induces ER stress is possibly through a direct interaction with the ER or an indirect interaction by ROS-mediated signaling. Further studies are warranted to examine the underlying mechanisms of mitochondrial ROS-mediated ER stress.

Taken together, we found that mitochondrial fragmentation occurs in PASMCs, which contributes to PASMC dysfunction through enhancement of ER stress in hypoxic rats. Inhibition of mitochondrial fragmentation attenuated ER stress and improved PASMC function, while inhibition of ER stress improved PASMC function without improvement of mitochondrial morphology in hypoxic rats. In addition, we found that mitochondrial fragmentation-induced ER stress is dependent on mitochondrial ROS. These results suggested that mitochondrial morphology plays an important role in the induction of PASMC dysfunction and could be a potential target in the treatment of PAH.

## MATERIALS AND METHODS

### Animal models of PAH

All animal experiments were reviewed and approved by the Animal Care and Use Committee of our university. Male Sprague-Dawley (SD) rats (150-200 g each) were used for the establishment of PAH models. Hypoxic rats were exposed to hypoxia (10% O_2_) for 8 h/d in a custom-developed hypoxia chamber for 4 weeks as described previously [13]. Normoxic rats underwent the same procedure but without hypoxia. Rats were intraperitoneally injected with Mdivi-1 (dissolved in 1 mL saline with 1‰ DMSO, 2.4 mg/kg/d, 4 weeks) or vehicle (1 mL saline with 1‰ DMSO) to study the effects of Mdivi-1 on pulmonary artery function. A total of 48 rats were used (8 rats for each group) in this study.

### Hemodynamic index detection

Rats in all groups received external jugular vein catheterization after anesthesia by continuous administration with 1.5%–2% isoflurane in 100% oxygen. A PowerLab/4SP biological signal acquisition and analysis system was applied to assess pulmonary pressure, which was detected by a sensor on the terminal of catheter as described previously [13]. Data were collected and mPAP was calculated. Cardiac output was detected using thermodilution and data were handled by a cardiac output detector (AD Instruments). PVR was calculated as the ratio of mPAP to cardiac output.

### Functional assessment of pulmonary artery

The pulmonary arteries were carefully excised and cut into ring segments (1 mm long) as described previously [14]. Briefly, the contractile force was detected using a temperature-controlled myograph (model 610M, Danish Myo Technology) and incubated in physiological saline solution (PSS). After a 40 min equilibration, PSS with high KCl (60 mM) was used to test the viability of vascular smooth muscle. For detection of vasodilation, pulmonary rings were pre-contracted with phenylephrine (PE) (10 μM). Endothelium-dependent vasodilation evoked by cumulative ACh (10^−10^ to 10^−5^) and endothelium-independent vasodilation evoked by cumulative SNP (10^−10^ to 10^−5^ M) were detected and expressed as the percentage of PE-induced contractile force. For detection of vasoconstriction, cumulative PE (10^−9^ to 10^−5^ M) was used.

### Transmission electron microscopy

The pulmonary arteries were excised and fixed in an electron microscopy fixation buffer. Transmission electron microscopy was performed as described previously [15]. The samples were visualized using a Hitachi microscope (H7500 TEM, Japan). Mitochondrial size and number were measured using the open-source image analysis program ImageJ (NIH).

### Isolation and culture of PASMCs

PASMC obtained from medial smooth muscle layer of pulmonary arteries were isolated as described previously [13]. PASMCs were cultured in DMEM. primary cultures created, grown to about 70% confluence, and maintained until 3-5 passage. PASMCs were exposed to hypoxic conditions (5% O_2_) for 24 h.

### Confocal imaging

An inverted confocal microscope (Zeiss LSM 800) was used for imaging. For detection of mitochondrial ROS, mitoSOX (5 μM) was loaded for 20 min. MitoSOX fluorescence was excited at 488 nm and the emission was collected at 540-625 nm. The mitochondrial network was monitored using mitoTracker Green (0.5 μM). Mitochondrial fluorescence using mitoTracker was reported by excitation at 488 nm and collecting the emission at >500 nm. All experiments were performed at room temperature (22–25° C).

### Western blotting

Protein expression was measured using western blotting. Briefly, the immunoblots were probed with anti-mfn1 (ab221661, Abcam), anti-mfn2 (ab205236, Abcam), anti-OPA1 (ab157457, Abcam), anti-Drp1 (ab184247, Abcam), anti-p-Drp1 (Ser637) (ab193216, Abcam), anti-p-Drp1 (ser616) (#3455, Cell Signaling Technology), anti-fis1 (10956-1-AP, Proteintech), anti-C/EBP homologous protein (CHOP) (#2895, Cell Signaling Technology), anti-myosin light chain (MLC) (#3672, Cell Signaling Technology), anti-p-MLC (#3675, Cell Signaling Technology), or anti-β-actin (ab8227, Abcam) overnight at 4° C followed by incubation with the corresponding secondary antibodies at room temperature for 1 h. The blots were visualized with ECL-plus reagent (SignalFire, USA).

### Statistical analysis

All values are presented as mean ± SEM. Data were compared with one-way ANOVA or two-way ANOVA, with all ANOVA tests followed by an unpaired t-test, as appropriate. Bonferroni’s correction for multiple comparisons was used. Differences were considered significant when *p* <0.05.
